# Reel Versus Twiddler Syndrome in a Patient With a Pacemaker: A Case Report of Iatrogenic Manipulation

**DOI:** 10.7759/cureus.65758

**Published:** 2024-07-30

**Authors:** Fernando de la Guia-Galipienso, Marisa de la Guia-Fayos, Miguel Angel Lopez-Aranda, Jose Manuel Simon-Machi, Aurelio Quesada-Dorador

**Affiliations:** 1 Cardiology, Hospital Clinica Benidorm (HCB), Benidorm, ESP; 2 Pathology, School of Medicine, Catholic University of Valencia San Vicente Mártir, Valencia, ESP; 3 Cardiology, Policlinica Glorieta Denia, Denia, ESP; 4 Pathology, Universitätsmedizin Neumarkt a. M. Campus Hamburg (UMCH), Hamburg, DEU

**Keywords:** pacemaker complication, pacemaker lead displacement, twiddler's syndrome, reel syndrome, cardiac pacemaker

## Abstract

Twiddler syndrome is a rare entity in which patient manipulation causes lead dislocation with lead retraction. Reel syndrome, on the other hand, is observed after external manipulation, intentional or unintentional, in which the leads move along their transverse axis and is considered a variant of Twiddler syndrome. We present the clinical case of a 91-year-old female who, after pacemaker implantation, presented with dizziness and chest discomfort following constant manipulation of the pacemaker, resulting in complete retraction of the lead tip into the pouch, which is unusual in the literature to date.

## Introduction

Both Twiddler and Reel syndromes are rare complications associated with transvenous pacemakers and implantable cardioverter-defibrillators [1,2], caused by pacemaker dislodgement due to patient-induced manipulation (conscious or unconscious), and coiling and retraction of the lead. Specifically, Twiddler syndrome is defined as a pacemaker malfunction in which the lead is dislodged by physical manipulation, resulting in coiling of the lead due to rotation of the generator on its longitudinal axis [3]. Conversely, Reel syndrome is a variant of Twiddler syndrome in which the device rotates on its transverse axis instead [4]. The electrodes coil near or around the generator and retract away from the heart, resulting in pacemaker malfunction, although there is usually no damage to the leads themselves. Also of note is Ratchet syndrome, in which the lead appears to be wrapped around the pulse generator, similar to Reel syndrome, but only one lead is involved [5,6]. The incidence in the most important published series is low (0.6%-1.8%) [7]. These syndromes are not easily differentiated, and diagnosing the specific cause of pacemaker malfunction can be challenging. We present the case of a patient with a suspected triggering factor for one of these syndromes and some unexpected findings.

## Case presentation

A 91-year-old female underwent implantation of a single-chamber pacemaker (Medtronic) in the left hemithorax for a complete atrioventricular block with recurrent syncope through the left subclavian vein. Due to the patient's age, it was decided to implant only a ventricular lead. Her medical history included chronic renal disease (stage 3) (glomerular filtration rate (GFR): 30-35 mL/minute/1.73 m^2^) for nephroangiosclerosis with controls in the nephrology unit with conservative therapy, hypertension, hypercholesterolemia, hyperuricemia, obesity grade I (BMI: 30.5 kg/m^2^), and preserved mental acuity with no clinical signs of dementia. She was independent in her daily activities.

**Figure 1:**
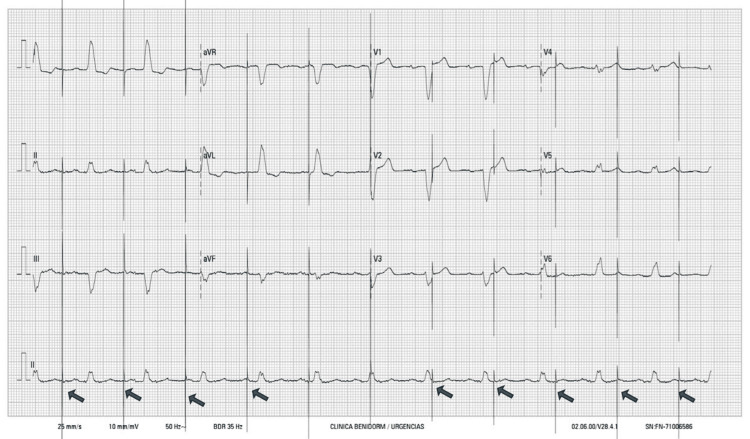
Emergency ECG Absence of detection and ventricular stimulation (arrows), suggesting pacemaker malfunction. ECG: electrocardiogram

**Figure 2:**
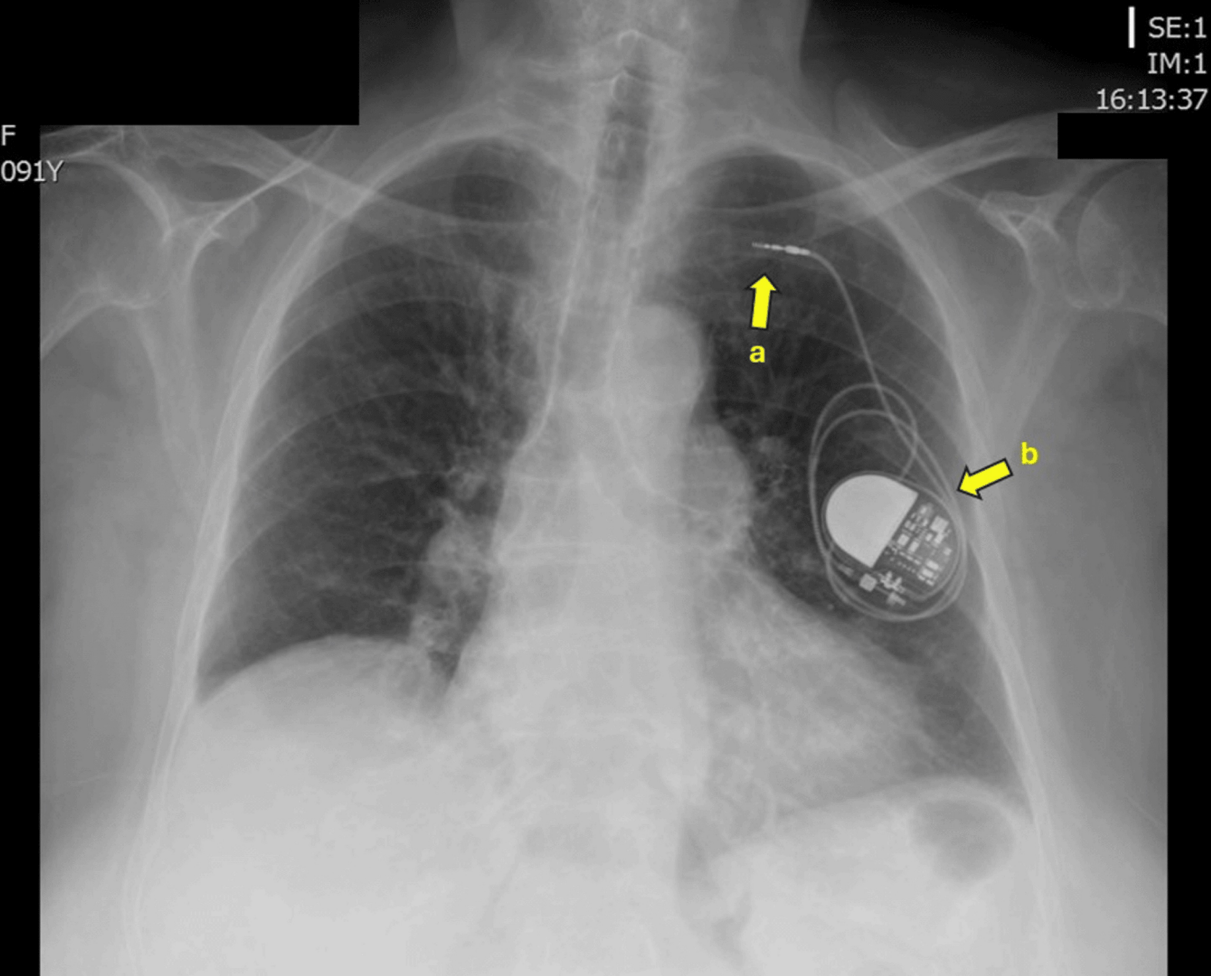
Chest X-ray (anteroposterior) Chest X-ray (anteroposterior) showing dislocation of the electrode in the venous system (a) with the pacemaker lead fully retracted into the pacemaker pocket (b).

Three weeks after pacemaker implantation, the patient presented to the emergency department with dizziness and chest discomfort. She reported an unpleasant rhythmic sensation around the implant and complained of involuntary movements of the entire left side of the chest and reflex activity of the left arm, which was found to be related to pacemaker stimulation of the chest. A 12-lead electrocardiogram (ECG) was performed and showed that the ventricle was not detected or recorded, suggesting a pacemaker malfunction (Figure 1). The device interrogation revealed no capture in the threshold from a few days before. A chest X-ray was performed; the lead was fully retracted into the device pocket (Figure 2). The patient felt uncomfortable with the device because it was implanted very superficially, rubbed against her clothes, and caused her pain, so she constantly touched and manipulated the area of the pacemaker implant. These findings were consistent with Reel syndrome, in which the leads are usually not damaged but retracted into the venous system (superior vena cava, brachiocephalic vein, or left subclavian vein) toward the pacemaker pulse generator. In this case, the tip of the lead was fully retracted into the pouch of the device, which is not common in clinical practice and has usually not been reported in previous cases.

**Figure 3:**
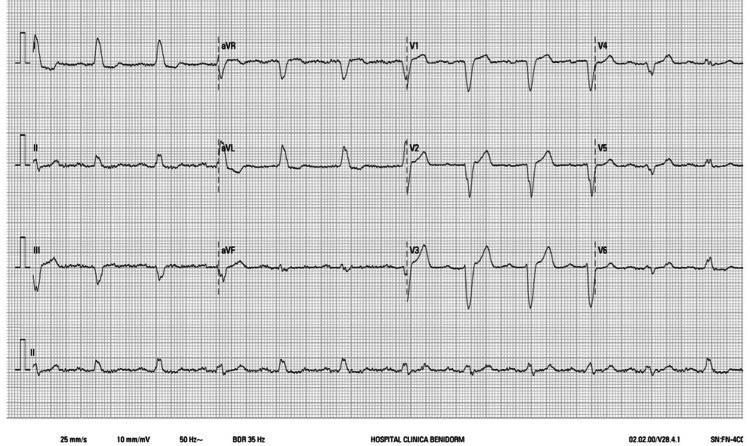
ECG after reintervention Sinus rhythm in the absence of pacemaker stimulation. ECG: electrocardiogram

**Figure 4:**
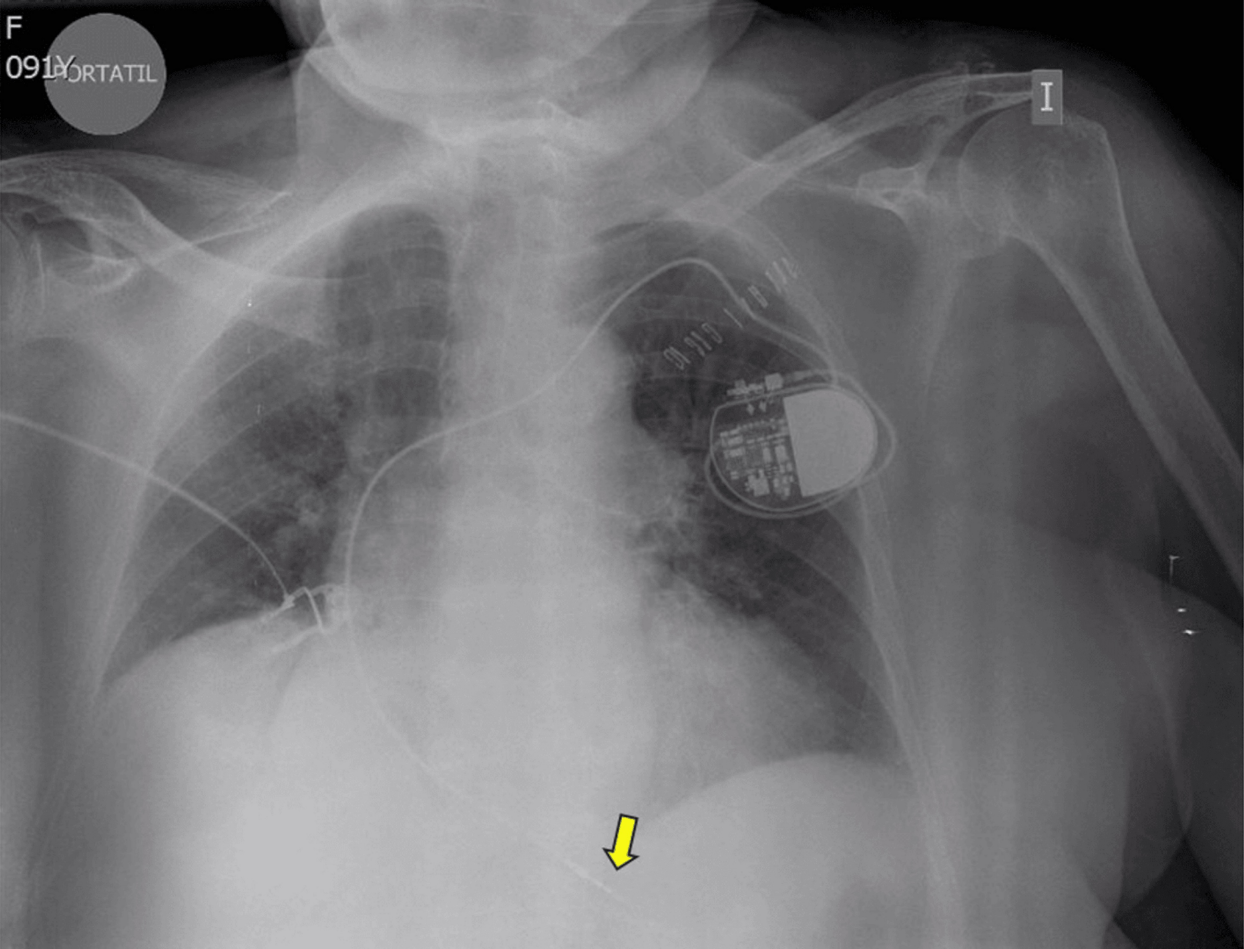
Anteroposterior chest X-ray after reintervention The adequately implanted lead can be seen in the right ventricular apex (arrow).

After confirming pacemaker dislocation, surgery was scheduled, using prophylactic intravenous antibiotic therapy following the usual protocol in our hospital (gentamicin 80 mg and cefazolin 1 g). The surgical wound was opened under local anesthesia; when opened, the device was found to be very shallow due to manipulation by the patient. The generator was displaced on its transverse axis, and as mentioned above, the electrode's tip was found within the device pocket. The stitches were tied tightly around the stitch sleeves. The lack of fixation of the device to the adjacent tissues, together with the manipulation of the patient, influenced this mechanism, which is why the device and electrodes were fixed more firmly with non-absorbable suture to the muscle fascia, placing several stitches. A new 58 cm lead was implanted without incident. The generator was secured to the underlying pectoral muscle with great care taken to immobilize the device within the pocket using a staystitch and to close the pocket around the device and lead. The patient was discharged without complication. One week later, she returned for a checkup. The surgical wound was in good condition. The pacemaker was checked, showing ventricular pacing of 5.5%, R-wave sensing thresholds of 11.20-15.68 Mv, impedance of 425 Ohms, and stimulation threshold of 0.50 V at 0.21 ms. A new ECG (Figure 3) and chest X-ray (Figure 4) were performed, showing sinus rhythm with first-degree atrioventricular block and no ventricular pacing, with the pacing lead correctly positioned in the apex of the right ventricle.

Regarding the cause of the pacemaker displacement, we confirmed that the device had been implanted rather superficially during the first procedure. There was a displacement of the generator from the implantation pocket to a lower area, mainly due to the manipulation, causing pain and discomfort; this fact eventually accelerated the patient's manipulation. This displacement facilitated the rotation of the generator and the consequent displacement of the lead.

## Discussion

Several types of mechanical pacemaker lead dislocation dysfunction syndromes have been described. The main difference between them is the mechanism causing the dislocation [7]. Both Twiddler and Reel syndromes can cause symptoms related to pacemaker malfunction (arrhythmias, dizziness, and syncope). One of the most common and typical symptoms leading to consultation is the presence of phrenic nerve stimulation by the dislocated leads, resulting in rhythmic diaphragmatic contractions. Most of these cases occur in the first year after implantation. Risk factors include female sex, advanced age, obesity, psychiatric illness, and device-pocket mismatch (i.e., the small size of the implanted device relative to its pocket). Chest X-ray and ECG confirm the diagnosis, with the presence of lead displacement and pacing/sensing dysfunction, respectively [8].

In our case, the chest X-ray showed the pacemaker lead coiling around the pulse generator (the "Reel syndrome"), with rotation of the pulse generator on its transverse axis [9,10]. A chest X-ray is required to diagnose and differentiate these entities. Reel syndrome usually does not damage the lead(s) and occurs within one month of implantation. Therefore, it is not usually necessary to replace the lead, unlike Twiddler syndrome, where extreme tangling of the leads can be seen, often leading to the decision to replace the leads. Our findings were that the lead tip had been retracted all the way into the pocket, which is unusual in the literature to date.

One of the issues that raises questions about the implantation of endovenous devices is the potential influence of venous access on the long-term function of the pacing leads. A study of 409 patients highlighted the use of axillary puncture as the access route of choice due to its lower risk of long-term lead dysfunction and its success rate in achieving venous access, which is analogous to subclavian puncture, the most common access due to its simplicity and high success rate [11].

## Conclusions

Reel syndrome is a relatively rare complication of pacemaker or defibrillator implantation. Patients should be informed of the risks associated with manipulating the generator, as anatomical and physical conditions or curiosity can lead to serious or even fatal complications. However, in some cases, the patient may completely deny that the device is being manipulated; it is possible that it is being manipulated unconsciously or even by other people. In our case, the patient manipulated it voluntarily because it was implanted very superficially and caused her daily discomfort. To avoid Reel syndrome, it may be useful to create a tight pocket, suture the cable sleeves firmly to the bottom of the pocket, and fix the generator to the fascia.
